# Role of Cytokines in Experimental and Human Visceral Leishmaniasis

**DOI:** 10.3389/fcimb.2021.624009

**Published:** 2021-02-18

**Authors:** Mukesh Samant, Utkarsha Sahu, Satish Chandra Pandey, Prashant Khare

**Affiliations:** ^1^ Cell and Molecular Biology Laboratory, Department of Zoology, Kumaun University, Almora, India; ^2^ Department of Microbiology, All India Institute of Medical Sciences, Bhopal, India

**Keywords:** visceral leishmaniasis, animal models, human, cytokine response, immunotherapy

## Abstract

Visceral Leishmaniasis (VL) is the most fatal form of disease leishmaniasis. To date, there are no effective prophylactic measures and therapeutics available against VL. Recently, new immunotherapy-based approaches have been established for the management of VL. Cytokines, which are predominantly produced by helper T cells (Th) and macrophages, have received great attention that could be an effective immunotherapeutic approach for the treatment of human VL. Cytokines play a key role in forming the host immune response and in managing the formation of protective and non-protective immunities during infection. Furthermore, immune response mediated through different cytokines varies from different host or animal models. Various cytokines viz. IFN-γ, IL-2, IL-12, and TNF-α play an important role during protection, while some other cytokines viz. IL-10, IL-6, IL-17, TGF-β, and others are associated with disease progression. Therefore, comprehensive knowledge of cytokine response and their interaction with various immune cells is very crucial to determine appropriate immunotherapies for VL. Here, we have discussed the role of cytokines involved in VL disease progression or host protection in different animal models and humans that will determine the clinical outcome of VL and open the path for the development of rapid and accurate diagnostic tools as well as therapeutic interventions against VL.

## Introduction

Visceral leishmaniasis (VL) is mainly caused by three species *L. donovani*, *L. infantum*, and *L. chagasi.* These species are obligate intracellular parasites that mostly target the visceral organs of the host. The clinical manifestations of the disorder show the severity of the disease, which could be highly fatal in its advanced stage. VL is a vector-borne infection communicated by female sandflies; it is known as a disease of poor people because of its high prevalence in developing countries or malnourished populations around the world. It mainly affects the tropical and subtropical countries. Symptoms of VL include enlargement of the liver and spleen, fever, extreme weight loss, hypergammaglobulinemia, as well as the low count of RBCs, WBCs, and platelets. The parasite resides within the hostile environment of the macrophages of the spleen, liver, and bone marrow of the host. The immune system of the host is also compromised during disease progression. There are 50,000 to 90,000 new cases of VL reported worldwide annually, while the mortality rate in untreated VL patients is over 95% (https://www.who.int/news-room/fact-sheets/detail/leishmaniasis).

The establishment of VL infection is mainly associated with host immune response where T helper type 1 (Th1) and T helper type 2 (Th2) cytokines play an important role in VL (Saha et al., 2007; Mondal et al., 2010; Nylén and Gautam, 2010). The Th1 response is known to protect from VL infection, whereas the Th2 immune response is responsible for parasite growth and disease progression. Th1 type immune response is mainly associated with the production of interleukin (IL)-12, interferon-γ (IFN-γ), nitric oxide (NO), and reactive oxygen species (ROS) (Kaye and Scott, 2011), while the secretion of Interleukin (IL)-4, IL-10 and transforming growth factor-beta (TGF-β) is mainly associated with Th2 immune response. Several other cytokines viz IL-3, IL-5, IL-6, IL-8, IL-9, IL-13, IL-15, IL-18, IL-23, and IL-27 also play a critical role during disease progression or protection **(** **Table 1** **).** (Elliott et al., 1989; Barral-Netto et al., 1991; Milano et al., 2002; Chamizo et al., 2005; Murray et al., 2006b; Stäger et al., 2006; de Lima et al., 2007; Maroof and Kaye, 2008; Murray, 2008; Zhu et al., 2010; Menezes-Souza et al., 2011; Ghosh et al., 2013b; Hajilooi et al., 2015; Pérez-Cabezas et al., 2016; Quirino et al., 2016; Ramos et al., 2016; Costa and Gomes, 2020; Lamberet et al., 2020; Moravej et al., 2020)

**Table 1:** Role of various cytokines in experimental models and humans for the management of VL.

Host	Cytokine	Functions in leishmaniasis	Possible outcome	References
Mouse	IL-10	Inhibits the production of iNOS, IFN-γ, IL-12, and TNF-α production and suppresses parasite killing by inhibiting Th1 cellular response	Immunosuppression and disease progression	(Murphy et al., 2001; Murray et al., 2002; Mesquita et al., 2018)
	IL-12	Triggers parasite specific Th1 response and controls Th2 expansion and IL-4 production	Host protection	(Heinzel et al., 1995; Murray et al., 1997; Antoine et al., 2004)
	IFN-γ	Activate Th1 response	Host protection	(Tsagozis et al., 2005)
	IL-2	Induction of IFN-γ	Host protection	(Murray et al., 1992)
	TNF-α	Induces granuloma response and wound healing process	Host protection	(Murray, 2000)
	TGF-β	Shows marginal effect on the parasite load and IFN-γ dependent host resistance, shows the biphasic kinetics; promotes as well as inhibits the inflammation and impairs the rate of disease cure in murine models	Disease progression	(McCartney-Francis and Wahl, 1994; Wilson et al., 1998; Gantt et al., 2003; Murray et al., 2005)
	IL-17	Proinflammatory cytokine	Host protection	(Ghosh et al., 2013b)
	IL-23	Shows IL-12 independent protection against visceral infection	Host protection	(Murray, 2000)
	IL-27	Increases susceptibility and affects the IL-17/neutrophil influx axis	Disease progression	(Quirino et al., 2016)
	IL-33	Suppresses Th1 responses in the livers	Disease progression	(Rostan et al., 2013)
	IL-4	Induces humoral immune response	Disease progression	(Pérez-Cabezas et al., 2019)
	IL-6	Induces an immunosuppressive effect on parasite infected liver	Disease progression	(Murray, 2008).
	IL-3	Induces MCCP formation in bone marrow and spleen	Disease progression	(Saha et al., 2004)
	IL-18	Required for anti-parasite activity	Host protection	(Murray et al., 2006a)
	IL-13	Participates in granuloma assembly	Host protection	(Murray et al., 2006b)
Hamster	IL-12	Drives Th1 response and IFN-γ production	Host protection	(Melby et al., 2001)
	IFN-γ	Activates macrophages to release ROS.	Host protection	(Melby et al., 2001)
	TNF-α	Activates the macrophages to kill amastigotes	Host protection	(Melby et al., 2001)
	TGF-β	Suppresses expression of inducible NO synthase and IFN-γ, and suppresses Th1 and Th2 cell development.	Disease progression	(Gantt et al., 2003)
	IL-4	Deactivation of macrophages and suppression of Th1 cells	Disease progression	(Samant et al., 2009)
	IL-10	Deactivation of macrophages and suppression of Th1 cells	Disease progression	(Samant et al., 2009)
Canine	IFN-γ	Involved in the activation of macrophages and the killing of the intracellular amastigotes in collaboration with TNF-α	Host protection	(Vouldoukis et al., 1996)
	IL-2	Activation of iNOS	Host protection	(Vouldoukis et al., 1996)
	IL-12 and IL-15	Upregulate T-bet expression and downregulate the expression of programmed cell death protein-1 (PD-1) in lymphocytes.	Host protection	(Costa and Gomes, 2020)
	IL-6	Hypergammaglobulinaemia	Disease progression	(de Lima et al., 2007)
	TGF-β	Inhibits T-cell proliferation, M8 activation, iNOS expression, TNF-α and IFN-γ functions and acts synergistically with IL-10 in disease progression	Disease progression	(Dayakar et al., 2019)
Non-Human Primates	IFN-γ	Clearance of intracellular parasites	Host protection	(André et al., 2020)
Human	IL-8	Increases neutrophil infiltration in VL patients	Disease progression	(Barral-Netto et al., 1991)
	IL-10	Blocks the activation of Th1 cells by down-regulating the production of IFN-γ and IL-12	Disease progression	(Bogdan et al., 1991)
	IL-12	Restores IFN-γ production and cytotoxic responses in visceral leishmaniasis.	Host protection	(Bacellar et al., 1996)
	IL-4	Inhibits human macrophage activation by TNF, GMCSF, and IL-3, Produces from PBMCs of cured VL patients in response to *L. donovani* crude or purified gp63 antigen stimulation	Both protection and disease progression	(Ho et al., 1992; Kurtzhals et al., 1994)
	IFN-γ	Activates macrophages and monocytes to release oxygen radicals and TNF-α, IL-l, and IL-6 secretion	Host protection	(Hart et al., 1989)
	TGF-β	Macrophage deactivation, suppress healing responses and avoid host parasite clearance	Disease progression	(Bogdan et al., 1991)
	TNF-α	Activation of effector immune response	Host protection	(Zwingenberger et al., 1991)
	IL-6	Inhibits production of TNF-α in the early stage of infection	Disease progression	(Costa et al., 2013; Dos Santos et al., 2016)
	IL-17	Affects neutrophils function, reduces apoptosis, induces the production of pro-inflammatory cytokines and tissue damaging molecules at inflammatory foci	Disease progression	(Dragon et al., 2008)
	IL-27	Increases the Th1 response and dampens the production of IL-17 mediated neutrophil infiltration	Host protection	(Quirino et al., 2016)
	IL-1	Impaired production of IL-1 from human PBMCs with *L. donovani-*antigen stimulation and successful therapy recovers IL-1 levels	Host protection	(Ho et al., 1992)
	IL-3	Enhances oxidative burst stimulation and secretion of TNF-α in human macrophages, thus protects parasite replication	Host protection	(Elliott et al., 1989)
	IL-15	Increases the IL-12 and reduces the IL-4 secretion	Host protection	(Milano et al., 2002)

Recent reports indicate the immune response against VL behaves differently in various organisms (Dayakar et al., 2019). During the past few decades, most of the studies have been done in the mouse model of VL. The murine model was broadly used for the study of the host immune response against VL infection (Murphy et al., 2001; Murray, 2001; Kaye et al., 2004; Sakthianandeswaren et al., 2009). Mice having different genetic backgrounds showed different immune responses. For example, BALB/c and C57BL**/**6 mice are susceptible, while SV/129 is considered resistant. However, the mouse model does not appear to exhibit a high susceptibility to VL infection since intravenous injection of visceral *Leishmania* results in a self-healing chronic infection. In addition, cytokine phenotypes exhibited in the mouse model by viscerotropic *Leishmania* are not characteristic of a Th2-type response (Papadopoulou et al., 2002). Apart from mice, the hamster model for VL infection is widely accepted for immunological studies (Melby et al., 2001; Garg and Dube, 2006; Aslan et al., 2013). The cytokine response in the hamster is pretty much similar to humans against VL infection. Currently, the Syrian Golden hamster is accepted worldwide to test various prophylactic and therapeutic measures. A major drawback of visceral models is that only large doses of antimony in the prime visceral disease model will eliminate known lesions (the hamster model). The model has also been used in vaccination research (Sharma et al., 2003; Sharma et al., 2004). However, the molecular mechanisms for this high degree of vulnerability are uncertain, and there are limited immunological studies applicable to this model due to the lack of available reagents.

Since all rodent models have the disadvantage of having different drug metabolisms and pharmacokinetics from humans, secondary testing in higher models such as dogs and monkeys with responses similar to humans will further validate primary screening and help in selecting the most promising molecules/epitopes for the development of a vaccine. A canine model of VL, such as dog, is now being considered for secondary testing of novel drug molecules and vaccine candidates against VL infection (Alvar et al., 2004; Baneth et al., 2008). Dogs share a highly similar immune response to humans. They are the major reservoir in the Middle East, the Mediterranean region, and South America. However, the nature of the infection is quite unpredictable, which has been a serious concern in the creation of canine VL experimental models but seems to demonstrate the spectrum of clinical responses seen during natural infections (Garg and Dube, 2006). The non-human primate (NHP) models are now being used to study the immunological responses and to validate host-parasite interactions against VL and considered as a model for tertiary testing due to their similarities to humans with respect to physiological and immunological characteristics (Alvar et al., 2004; Baneth et al., 2008). In VL infection, non-human primates *Aotus trivirgatus* (owl monkeys) and *Saimiri sciureus* (Squirrel monkeys) are currently being used in the New and Old World (André et al., 2020). They have shown all clinical and immunopathological characteristics found in human VL (Singh and Sivakumar, 2003). The Asian rhesus macaques (*Macacamulatta*) and the Indian langurs are also susceptible to VL infection and have also been used for preclinical studies (Misra et al., 2001). However, the disadvantage is that the primates are costly laboratory animals that are hard to procure and treat and also immunological black boxes. In human visceral leishmaniasis, the Th1/Th2 cytokine balance has been well documented either for pathogenesis or host protection (Dayakar et al., 2019). During the parasitic disease, various micro-RNAs (miRNAs) play an important role in the proliferation, differentiation, and production of CD4**^+^** T cells (Arora et al., 2017; Chapman et al., 2017; Budak et al., 2018). However, the differentiation of naive CD4**^+^** T cells into Th1/2 cytokines is strictly regulated in leishmaniasis, *L. donovani *infection can induce differential miRNA expression in CD4+ T cells and macrophages (Tiwari et al., 2017; Kumar et al., 2020). Further, silencing the microRNA-21 (miR-21) resulted in an augmented induction of IL-12 in *L. donovani*-infected mouse dendritic cells (Gannavaram et al., 2019). *L. donovani* infection also downregulates microRNA-122 (miR-122) and genes involved in cholesterol biosynthesis in infected mouse livers (Ghosh et al., 2013a). The differentiation of naive CD4**^+^** T cells to the Th1/2 phenotype includes various pathways viz. the JAK-STAT pathway, notch protein-associated pathway, and MAPK signaling pathways (Radtke et al., 2010). Notch 3, JAK1/2, ZAP70, STAT1, and STAT4 are the transcription factors involved in these pathways, which helps CD4**^+^** T cells to differentiate into Th1 subtype, while Th2 phenotypes differentiation occurs using Notch 1/2, STAT5, STAT6, GATA3 transcription factors (Radtke et al., 2010). It has been well documented that the upregulated miRNAs (miR-7a-1-3p, miR-574-5p, miR-690, miR-7017-5p, and miR-7235-5p) target the notch 3 gene. Notch 3 has a prominent role in the immune response of Th1, such as the development of IFN-γ. However, miRNA inhibits the notch 3 gene in leishmaniasis and impairs the development of IFN-γ, thereby providing an ideal environment for parasite survival. Moreover, STAT 4 and STAT1 have a main function in JAK-STAT signalling for IFN-γ production, and miR-574 and miR-6994-5p regulate these genes. In addition, it has also been stated that downregulated miRNAs dominated the differentiation of naive CD4**^+^** T cells into the Th2 phenotype. IL-4, a major Th2 cytokine secreted by CD4**^+^** T cells, is the target of miR-340-5p. IL-2 and IL-13 are major CD4**^+^** T cell-secreted Th2 cytokines (Murray et al., 1993; Murray et al., 2006b), but their regulation is carried out by downregulated miRNA (miR-3473f and let 7j). It has been also reported that downregulated miRNAs control the differentiation of naive CD4**+** T cell to Th2 phenotype. Further research is therefore required to understand their function in *Leishmania*-induced Th2 immune responses. The STAT 5 and STAT 6 genes are targeted by miRNA-93-3p and 486a-3p, and act as the major transcription factors in Th2 differentiation. As these two miRNAs were found to be downregulated in infected CD4**^+^** T cells, it is hypothesized that these miRNAs may have a potential role in the management of T cell proliferation, differentiation and Th1/Th2 dichotomy in *Leishmania* pathogenesis. Notch 1/2 and GATA 3 are prominent genes that are involved in the differentiation of CD4**^+^** T cells from the Th2 phenotype (Gilmour and Lavender, 2008). It is further concluded that the above transcription factors are targeted by downregulated miRNAs (miR-93-3p, let 7j, 486a-3p, and miR-3473f) and the downregulation of these miRNAs contribute to the differentiation of CD4**^+^** T cells into the Th2 phenotype in the case of *Leishmania* infection (Kumar et al., 2020).

**Figure 1:**
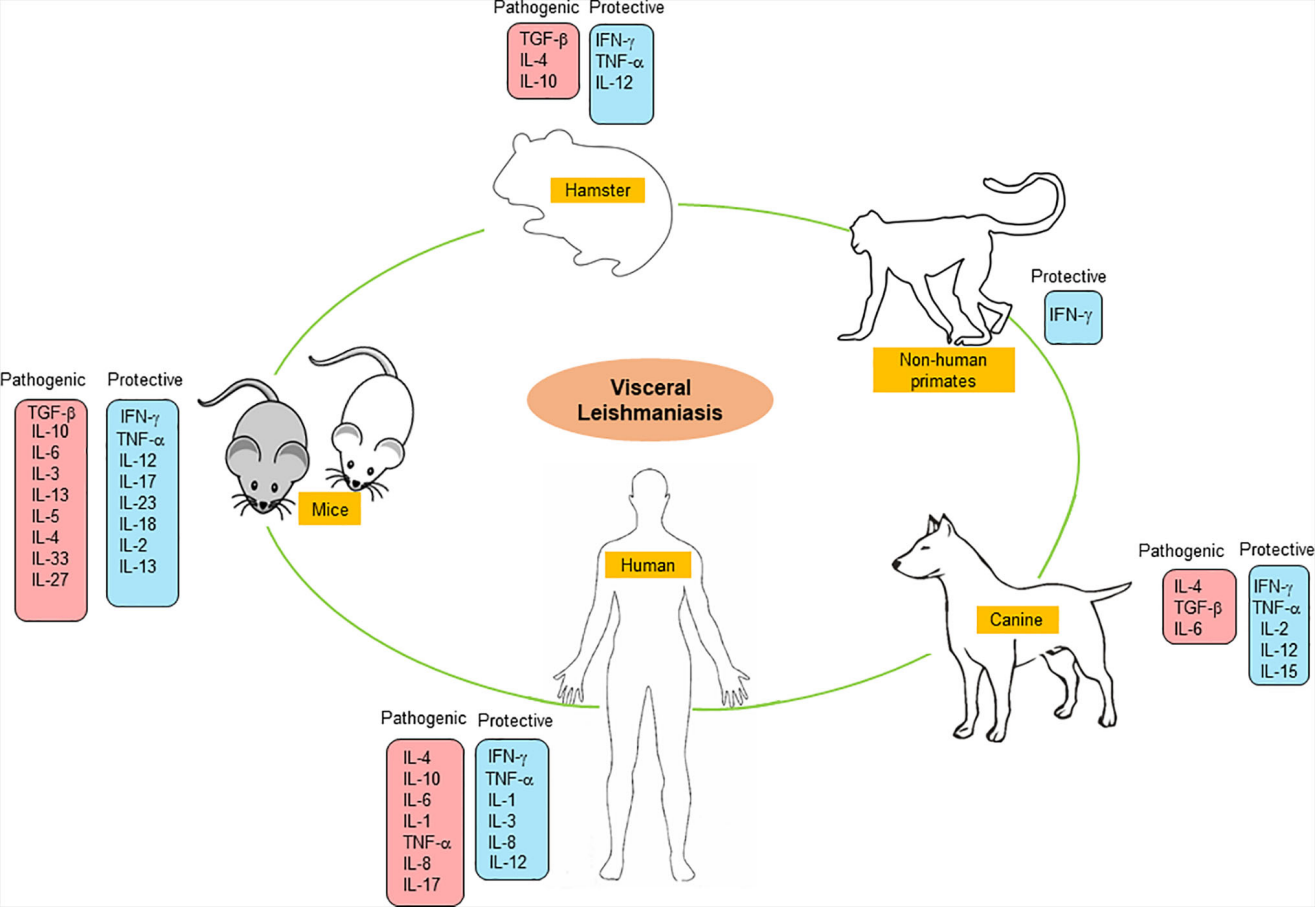
Pathogenic and protective cytokine response in experimental and human VL.

Here, we have summarized the cytokine-based immune response events occurring in various experimental animal models e.g., mouse, hamster, canine, non-primates, and human against VL infection **(** **Figure 1** **).** This review highlights the in-depth analysis of various cytokines in disease progression and host protection that could be used for the development of an effective immune-therapeutic tool against VL.

## Cytokine Response in Various Animal Models and in Human Against VL

### Cytokine Response in Mice

**Figure 2:**
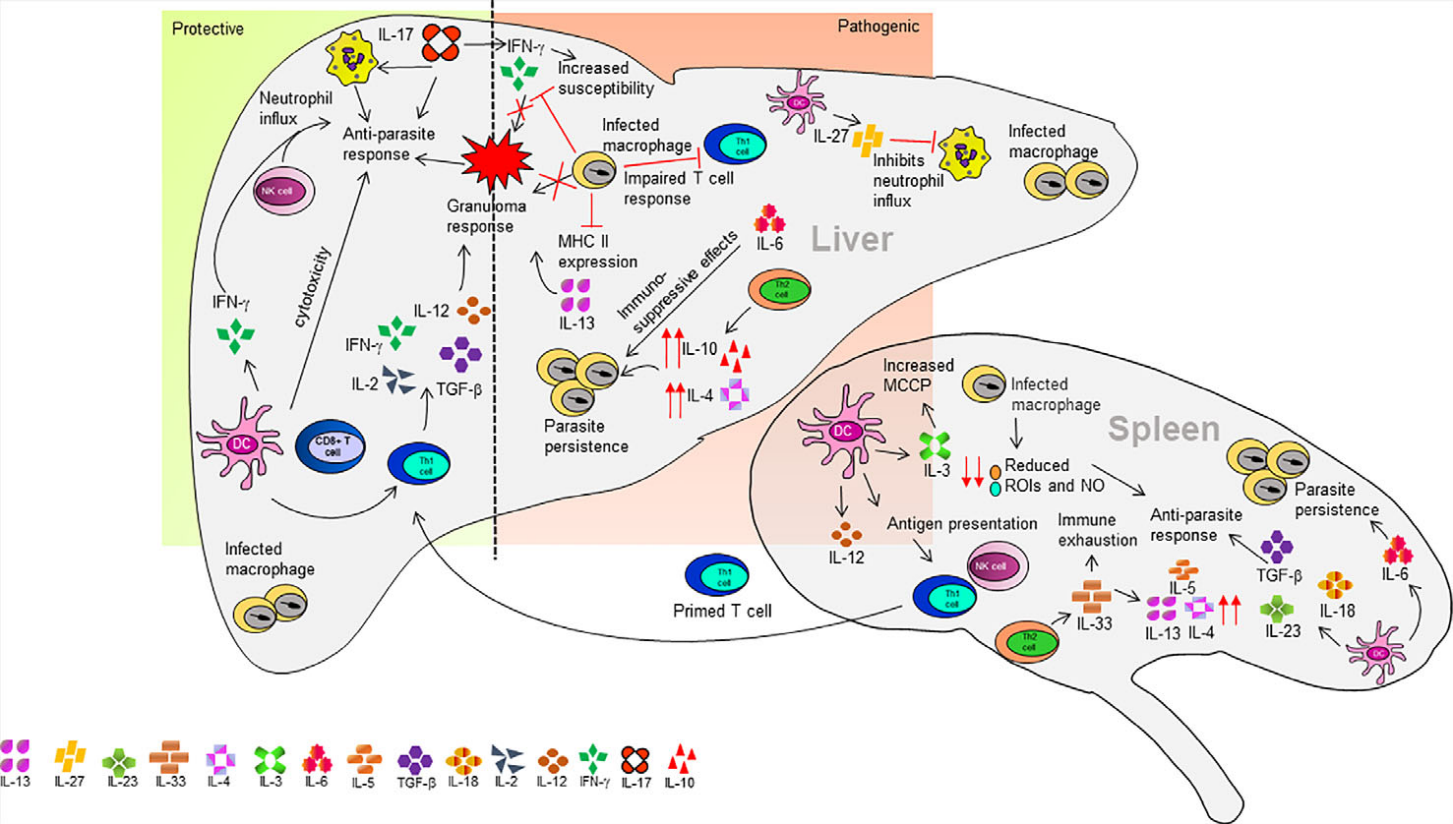
Organ-specific cytokine response in a mouse model of VL.

The mouse model of VL is an extensively studied disease model. VL infection in mice is established through metacyclic promastigotes or amastigotes form of *L. infantum* and *L. donovani* (Murray, 2001; Kaye et al., 2004). Mice having different genetic backgrounds showed susceptibility or resistance against VL infection. Various studies investigated the evasion of host immune response by *Leishmania* parasites and genes associated with innate and acquired immunity against VL (Kirkpatrick et al., 1985; Kaye et al., 2004; Wegner et al., 2004; Lipoldova and Demant, 2006; Marquis and Gros, 2007; Stanley and Engwerda, 2007; Huynh and Andrews, 2008). For example, in the resistant mouse strains CBA, the functional involvement of Slc11a1 induced macrophage activation. The slc11a1 gene encodes a membrane protein on macrophages and increases nitric oxide (NO) production mediated by the induction of the inducible nitric oxide synthases (iNOS) and finally inhibiting the parasite growth (Blackwell et al., 2001). These mice are resistant to the early growth of the *Leishmania* parasite. Similarly, SV/129 mice strain is considered to be resistant. This may be due to the significant upregulation of IL-10 producing CD4^+^ T cells, which could indicate an effective anti-parasitic response (Pérez-Cabezas et al., 2019). On contrary, the susceptible strains like BALB/c and C57BL/6 have non-functional Slc11a1gene and thus early growth of *Leishmania* is not inhibited in them (Kaye et al., 2004). The development of susceptibility and resistance to the *Leishmania* parasite also depends on the development of Th1 and Th2 type cellular response. During *L. infantum* and *L. donovani* infection in murine models, the Th1/Th2 paradigm is not influential like in other models in which the Th1 response is suppressed by TGF-β and IL-10 (Tripathi et al., 2007). The BALB/c and C57BL/6 mice are susceptible strains to *L. donovani* visceral infection. The self-healing C57BL/6 mouse displays an early Th1 response after infection, while the non-healing BALB/c mouse strain displays an early Th2 response leading to disease progression **(** **Figure 2** **)** (Garg and Dube, 2006).

In active VL, the membrane cholesterol of macrophages also plays a crucial role in promoting parasite internalization (Asad et al., 2015). Interestingly, *L. donovani* extracts membrane cholesterol from macrophages and disrupts lipid rafts, leading to their inability to stimulate T cells (Ghosh et al., 2012). The conformation of a large number of membrane proteins is considered to be dependent on membrane cholesterol. Cholesterol is reported to be essential in maintaining MHC-II protein conformation and peptide-MHC complex stability, whereas *Leishmania* parasites can alter membrane cholesterol and modify MHC-II protein conformation, leading to defective T-cell stimulation in leishmaniasis (Roy et al., 2016).

The BALB/c mice strain is the most explored mouse model in VL. The immune response to VL in BALB/c mice varies between the liver and spleen within the same mice. The spleen is an initial site for the production of cell-mediated immune response but finally becomes a site for parasite persistence causing splenomegaly, tissue damage, and immunocompromising the host (Faleiro et al., 2014). The parasites entering the spleen are driven out by marginal zone (MZ) macrophages and dendritic cells (DCs). However, to some extent, the DC acquired *Leishmania* antigens are derived from phagocytosis of infected macrophages or by their fragments in MZ (Bankoti and Stäger, 2012). The DCs retain parasite antigens at MZ and then migrate to periarteriolar lymphoid sheath (PALS) where they secrete IL-12 and present antigen to NK cells and T cells (Ato et al., 2006). The *L. infantum *infection induces IL-12 secretion by splenic DCs within the PALS but not macrophages at the MZ (Gorak et al., 1998). Also, the macrophages have reduced capacity for the generation of anti-leishmanial molecules like reactive oxygen intermediates (ROIs) and NO required for killing of the pathogen (Awasthi et al., 2004). The primed T cells from the spleen migrate to the liver and generate a granuloma response **(** **Figure 2** **)**. At the same time during granuloma formation, the fusion of infected macrophages to form multinucleated cells contributes to the production of inflammatory cytokine (Murray, 2001). The inflammatory environment of target organs influences parasite elimination or persistence (Rodrigues et al., 2016). The failure in generating an efficient granuloma response and low expression of MHC II on the macrophage surface and defect in the generation of anti-leishmanial molecules against the parasite all contribute to the failure of an immune response to eradicate VL (Stanley and Engwerda, 2007). Upon infection, the *Leishmania* parasites enter the liver and invade macrophages and DC. These cells start secreting chemokines like C-X-C motif chemokine ligand 10 (CXCL10), chemokine C-C motif ligand 2 (CCL2), and chemokine C-C motif ligand 3 (CCL3) for recruitment of granulocytes and monocytes (Gupta et al., 2009; Nieto et al., 2011). The persistence of parasite infection is mediated by the macrophages either by irregular antigen presentation or by upregulation on interfering prostaglandin (Nylén and Gautam, 2010). The infection of BALB/c mice with *L. donovani* parasite resulted in increased intercellular adhesion molecule 1 (ICAM-1) and B7-1 (CD80) expression by infected macrophages. While in C57BL/6 mice, no significant changes were observed in the level of ICAM-1 and CD80. Furthermore, *Leishmania* infected macrophages are not able to generate co-stimulatory signals to T- helper cells mediated by prostaglandins, and inhibition in the synthesis of prostaglandin recovered this defect (Saha et al., 1995). Even after the activation of the innate immune response during early-stage infection, the parasite persists in the liver in the absence of activated T cells and a trace amount of inflammatory cytokines. However, during the later stage of infection, the parasite burden decreases in the liver with the acquisition of granulomatous response (Stanley and Engwerda, 2007; Nieto et al., 2011). Few studies have suggested that before migrating into the liver, the T-cells are pre-activated in the spleen (Engwerda and Kaye, 2000). Once the activated T-cells reach the liver, they interact with infected DCs and secrete IL-12, thereby triggering a parasite-specific Th1 response (Antoine et al., 2004). The activated DCs can induce IFN-γ and natural killer (NK) cell cytotoxicity (Schleicher et al., 2007). On the contrary, IL-12 production is obstructed in parasite-infected macrophages, which leads to an impaired CD4**^+^** T cell response (Soong, 2008). Hence, the interaction of DCs, and parasite-specific CD4**^+^** T cells in the liver provides an appropriate inflammatory environment required for granuloma formation leading to acquired hepatic immune response associated with the production of IL-2, IL-12, tumor necrosis factor (TNF)-α, and IFN-γ (Murray et al., 1992; Wilson et al., 2005). *L. infantum* regulates its local environment by TGF-β activation (McMahon-Pratt and Alexander, 2004). In *L. infantum *infected BALB/c mice, the acquired resistance depends upon granuloma development (Nieto et al., 2011) and an increased granuloma maturation indicates successful vaccination for VL (Carter et al., 2007). Different cytokines play crucial roles in parasite killing by granuloma formation. IL-12 produced by Kupffer macrophages (liver resident macrophages) induces IFN-γ by lymphoid cells associated with granuloma enhancing the leishmanicidal property of Kupffer cells (Murray et al., 1992; Stanley and Engwerda, 2007). Both *L. infantum* and *L. donovani* inhibits the host macrophage response against IFN-γ (McMahon-Pratt and Alexander, 2004). One of the crucial factors for assembly and maturation of granuloma is TNF (Kaye et al., 2004). In addition, lymphotoxin-α, a TNF-associated cytokine, promotes leukocyte migration towards Kupffer cells (Engwerda et al., 2004). IL-13, a Th2 cytokine is responsible for granuloma assembly and IFN-γ secretion. In *L. donovani* infected IL-13**^–/–^** mice with BALB/c background, the granuloma assembly, and IFN-γ secretion was significantly impaired in the liver (Murray et al., 2006b). The CD8**^+^** T cells also play a crucial role in different models of VL comprising both secretions of chemokines, cytokines, and cytotoxic activity (Tsagozis et al., 2003). The CD8**^+^** T cells are involved in controlling VL infection in BALB/c mice, which are nonself-curing mice, as this is a T-cell immunodeficient strain that manifests systemic visceral leishmaniasis (Howard et al., 1980). In BALB/c mice, the CD8**^+^** T cells displayed cytotoxicity against cells displaying parasite antigens. This cytotoxic activity was mediated by both Fas/Fas pathway and perforin. They also upregulated the mRNA levels of TNF-α, IFN-γ, RANTES, and macrophage inflammatory protein-1a (MIP-1a), which plays a major part in anti-parasite function (Tsagozis et al., 2003). IL-27 is mainly secreted by the DC in mouse model of VL (Pérez-Cabezas et al., 2016). In *L. infantum* infected BALB/c mice, IL-27 was upregulated but not in resistant C57BL/6 mice (Pérez-Cabezas et al., 2016). The neutralization of IL-27 decreased IL-10 production, and administration of recombinant IL-27 (rIL-27) increased IL-27 in C57BL/6 mice as compared to control animals (Pérez-Cabezas et al., 2016). Furthermore, treatment of rIL-27 significantly reduced the level of IL-12 and IFN-γ in C57BL/6 mice. Neutralization of IL-27 in acutely infected BALB/c led to decreased parasite burdens and a transient increase in IFN-γ+ splenic T cells, while administration of IL-27 to C57BL/6 promoted parasite infection. The rIL-27 prevented neutrophil infiltration in the spleen of C57BL/6 mice. IL-27 neutralization increased IFN-γ producing CD4^+^ and CD8^+^ T cells, but rIL-27 did not affect cytokine profile in splenic T cells of C57BL/6 mice (Pérez-Cabezas et al., 2016). IL-27 mediates susceptibility to *L. infantum* infection in the murine model by suppressing the IL-17 driven neutrophil response. IL-27**^–/–^** mice displayed better parasite control and upregulated IL-17 leading to increased neutrophil influx. This in turn, aids the parasite control in the target organ. Thus, the secretion of IL-27 in *L. infantum* infection increases susceptibility and affects the IL-17/neutrophil influx axis (Quirino et al., 2016).

Both IL-18 and IL-23 are required for a late-acting anti-parasite response in the mouse model of VL (Murray et al., 2006a). The splenic DCs secrete IL-23 after the parasitic infection, which, along with other cytokines (TGF-β, IL-6, or IL-1β), promotes Th17 differentiation (Maroof and Kaye, 2008; Zhu et al., 2010). The IL-17-producing Th17 cells are crucial elements of anti-parasitic activity in the mouse model, however, their role is different in VL. IL-17 in synergy with IFN-γ promotes parasite clearance in C57BL/6 mice. The 17R**^–/–^** mice with C57BL/6 backbone has an increased susceptibility to *L. infantum* infection and reduced parasite clearance and infiltration of IFN-γ producing cells. This leads to increased IL-10 producing CD4**^+^** T cells. Also, IL-17 in association with IFN-γ mediates NO production in infected macrophages, thus performing the anti-parasite activity (Nascimento et al., 2015). The BALB/c model of *L. donovani* induced VL was utilized to study the potential of curdlan, (a gel-forming polysaccharide) against VL (Ghosh et al., 2013b). The curdlan treatment significantly increased the Th17 cytokines (IL-23 and IL-17) levels in mice (Ghosh et al., 2013b). Another compound astrakurkurone induces IL-17 and IFN-γ production in the BALB/c mouse model of VL (Mallick et al., 2016) suggesting a protective role of IL-17 in VL. On the contrary, IL-17 is also reported to increase susceptibility to murine VL by modulating IFN-γ response (Terrazas et al., 2016). Different studies have reported the role of IL-33 and ST2 receptor in VL. The ST2 deficient mice showed better parasite control in the liver by early Polymorphonuclear leukocytes (PMN) infiltration and polarized Th1 immune response. Whereas the addition of recombinant IL-33 in BALB/c mice leads to a repressed Th1 response (Rostan et al., 2013). Another study suggested the upregulation of IL-33 and ST2 in both BALB/c and in C57BL/6 infected with *L. donovani*. However, the level of expression in BALB/c mice was twice that of the C57BL/6 mice. Treatment with recombinant IL-33 increased the parasite burden in the mouse spleen. IL-33 treatment in BALB/c mice resulted in immune exhaustion and downregulation of splenic Th1 cytokines (IL-12 and IFN-γ) accompanied by an upregulation in Th2 cytokines (IL-13, IL-5, and IL-4). In IL-33**^–/–^** mice, the parasite load was reduced, and the Th1 response increased, as compared to the WT mice (Lamberet et al., 2020).

In self-curing mouse model C57BL/6, CD8**^+^** T lymphocytes do not appear to play a primary role in parasite control but the IFN-γ-mediated Th1 response plays a crucial role in parasite control and prevention of disease during *L. infantum* infection (Tsagozis et al., 2005). IL-6 is mainly produced by the antigen-presenting cells of the immune system. In *L. donovani* infected C57BL/6 mice, IL-6 was involved in pathogenesis. In IL-6**^–/–^** mice, the rate of parasite killing and infection control was higher as compared to WT mice. Also, an increased Th1 response was observed in IL-6 **^–/–^** mice (Murray, 2008). IL-6 thus induces an immunosuppressive effect on the parasite-infected liver and can be a potential target for therapeutic blockade (Murray, 2008). In another *L. donovani* infected C57BL/6 mice, IL-6 in association with IL-12 mediated protective immune response and independently regulated IL-10**^+^** CD4**^+^** T cell expansion (Stäger et al., 2006). During *Leishmaina* infection, the susceptible BALB/c mice displayed more IL-3 producing T cells than the resistant C57BL/6 mice (Saha et al., 1999). There is an increase in mast cell committed progenitors (MCCP) in the spleen and bone marrow during the parasite infection *via* IL-3 (Saha et al., 2004). These findings suggest that a similar mechanism is also reported in *L. donovani* infection and lower MCCP in resistant mice due to the downregulation of IL-3 (Saha et al., 2004).

IL-10 is a key immunosuppressive factor in VL. The study performed on wild type C57BL/6 mice and transgenic pMT-10 on C57BL/6 background mice infected with *L. donovani* parasite was used to demonstrate the involvement of increased IL-10 production in the early stage of VL infection rather than the later stage (Mesquita et al., 2018). Another study on IL-10 deficient BALB/c and C57BL/6 mouse model of VL (Murphy et al., 2001; Murray et al., 2002) showed that the BALB/c IL-10**^–/–^** and C57BL/6 IL-10**^–^** ^/^ **^–^** mice were resistant to *L. donovani* infection. The parasite burden in the liver was reduced by 10 folds while the production of IFN-γ and nitric oxide increased in BALB/c IL-10**^–^** ^/^ **^–^** mice. The susceptibility to *L. donovani* infection increased after *in vivo* treatment with IL-12 and IFN-γ neutralizing antibodies (Murphy et al., 2001). Also, treatment with an anti-IL-10 receptor (IL-10R) monoclonal antibody (mAb) promoted the killing of parasites by macrophages through the upregulation of IFN-γ, IL-12 protein, and iNOS synthesis (Murray et al., 2002). Altogether these results imply the immunosuppressive role of IL-10 in VL. Finally, IL-10 suppresses parasite killing by inhibiting the Th1 cellular response (Murphy et al., 2001; Murray et al., 2002).

A spontaneous recessive lymph node T cell (plt) mutation results in loss of functional chemokine (C-C motif) ligand 21 (CCL21) and chemokine (C-C motif) ligand 19 (CCL19) genes and an abnormally formed lymphoid T cell zone (Nakano and Gunn, 2001). Due to the lack of these chemokines in plt/plt mice, the migration of T cells gets tampered resulting in a reduced recruitment of DCs and naive T cells at secondary lymphoid organs (Förster et al., 1999; Gunn et al., 1999). A study performed on CCL21/19 plt/plt mice with C57BL/6 mice background showed increased susceptibility to *L. donovani* infection as compared to normal mice (Ato et al., 2006). The activation of DC is inadequate after infection, along with reduced migration of DC from MZ to PALS. Altogether, the tampered DC activation in plt/plt mice leads to increased susceptibility and upregulated IL-10 mRNA. In the liver, the effector CD4**^+^**T and CD8**^+^**T cell migration was delayed with granuloma formation indicating a crucial role of chemokines in DC and T cell-mediated defense against *L. donovani* infection (Ato et al., 2006). During the VL, neutrophils also perform a key role. These are the major leukocytic cell effectors of the innate immune response, as neutrophils are the first cells quickly deployed to the parasite inoculation site in the process of VL, where they perform an important role in the early identification and removal of the parasites (Oualha et al., 2019). Neutrophils exude lytic enzymes and nitric oxide, causing various pathogens to die, thereby engaging in phagocytosis, which is regulated by the Toll-like receptor family, opsonins, and lipopolysaccharides to kill microorganisms. BALB/c mice infected with *L. donovani* and deficient in neutrophils display a rise in the parasite burden from the spleen and bone marrow and a decrease in the development of liver granulomas, with a decrease in the synthesis of nitric oxide (Cecílio et al., 2014). In the absence of neutrophils, the immune response against the parasite is changed with elevated IL-10 and IL-4 in the serum and spleen and less in IFN generating CD4**^+^** T and CD8**^+^** T cells, indicating that the Th1-type immune response is impaired in the absence of neutrophils (McFarlane et al., 2008). Furthermore, *Leishmania* may utilize the neutrophil as an escape mechanism when phagocytised in non-lytic compartments that presents endoplasmic reticulum markers and are unable to integrate with lysosomal organelles. The genes lpg1 and lpg2, which express phosphoglycans of *Leishmania*, are directly implicated in the parasite’s ability to stay in these compartments, preventing their destruction and slowing the apoptosis of neutrophils to extend their cell existence (Gueirard et al., 2008).

The humoral immune response to VL is mainly associated with the IgG1 isotype and is mediated by IL-4  (Pérez-Cabezas et al., 2019). The IgG specific response to *L. infantum* was evaluated in three different strains of mice model e.g., BALB/c, C57BL/6, and SV/129 mice, Interestingly it was observed that SV/129 mice had higher serum IgG levels specific for *L. infantum* as compared to BALB/c and C57BL/6 mice during the early phase of infection but with time, the level of IgG remarkably reduced, which is an indication of parasite clearance in SV/129 mice. However, BALB/c and C57BL/6 mice showed a prevalent IgG1 response that increased with the course of infection  (Pérez-Cabezas et al., 2019). Further, it has also been reported that in the BALB/c mice model, IgM is responsible for disease exacerbation, and polyclonal B cell activation is an early and intrinsic characteristic of VL (Deak et al., 2010). Therefore, B-cell-derived immunoglobulins (IgM and IgG) potentially may contribute to disease and parasite persistence throughout infection. These findings also proved why SV/129 strain is resistant to VL while BALB/c and C57BL/6 are considered to be susceptible to *L. infantum* and *L. donovani* parasites.

### Cytokine Response in Hamster

The Syrian golden hamster is considered an appropriate model for VL studies due to high susceptibility—a similarity with human pathology and unaffected parasite growth in the liver, spleen, and bone marrow (Miao et al., 2019). The immunopathology of VL in hamsters includes increased parasite burden, splenomegaly, cachexia, and hypergammaglobulinemia (Melby et al., 2001; Garg and Dube, 2006; Aslan et al., 2013). The expression profile of different Th1 (IL-12, IFN-γ and, TNF-α) and Th2 cytokines (TGF-β, IL-4, and IL-10) in *L. donovani* infected hamsters were well documented. The reports suggested an increased mRNA profile of Th1 cytokines such as IFN-γ and IL-12 during the early phase of infection while, a lower IL-12 mRNA level after 7 days post-infection was observed close to IFN-γ level (Melby et al., 1998; Melby et al., 2001). The increased mRNA level of IFN-γ during disease progression correlated with defective macrophage effector function. The NOS activity in spleen and liver tissues of *L. donovani* infected hamsters was not detected at any time of infection. Thus, the loss of this crucial anti-parasite activity in hamsters explains its inability to restrict parasite growth and high susceptibility (Melby et al., 2001; Perez et al., 2006). In the case of the Th2 cytokine, the expression of IL-4 in infected hamsters was almost at basal level and the same as observed in uninfected hamsters (Melby et al., 1998). The splenic expression of macrophage deactivator Th2 cytokine IL-10 increased over the first 4 weeks of infection, indicating the role of IL-10 in disease progression (Melby et al., 2001). Altogether there was a suppression of Th1 response and elevation of Th2 response during *L. donovani* infection in the hamster. A similar observation was made by another study where NO synthesis was absent in the spleen and liver of the hamster. The mRNA level of IL-4 was absent while IL-10 occurred during the later phase of infection. The mRNA expression of TGF- β was at a basal level while the protein form increased during the later phase. This irregularity between mRNA and protein TGF- β was due to the presence of Cathepsin B in *L. infantum* and *L. donovani* that promoted the growth of parasites within macrophages and activated TGF-β (Somanna et al., 2002; Goto and Lindoso, 2004). It is evident from the above information that a Th2 dominant immune response is associated with VL infection in hamsters. Treatment with miltefosine in *Leishmania* infected hamster suppressed the Th2 response and enhanced Th1 cytokine production, resulting in the cure of the animals from disease (Gupta et al., 2012). VL infection in the hamster is also characterized by the diminishing of the proliferative response to *Leishmania* specific antigens (Loría-Cervera and Andrade-Narváez, 2014). The VL in hamsters is generally induced by injecting parasites intracardially, intravenously, and intra-peritoneally. However, none of them mimic the effects of the sandfly bite, the natural mode of transmission. As the major antigen-presenting cells (APCs), the macrophages are not only the shelter of *Leishmania* but also the primary cells engaged in *Leishmania* inhibition. It has been demonstrated that *in vitro* infection of macrophages by *Leishmania* makes them immune to apoptosis (Moore and Matlashewski, 1994). Further, it is also reported in *L. chagasi* infected hamsters, macrophage apoptosis was conferred during the early phase of the infection (Moore and Matlashewski, 1994). Apoptosis of macrophages, however, vanishes from both the liver and spleen as the infection continues to grow, indicating defense of macrophages by *Leishmania* infection (JAL, 2001). Macrophage-mediated immune suppression is documented to lead to increased growth of the parasite and to be associated with either impaired presentation of antigen, suppression of MHC class I and II molecule expression, or regulation by prostaglandin-like substances (Murray et al., 1986; Reiner, 1987; Saha et al., 1995). In hamsters infected with *L. donovani*, adherent splenic cells have been found to be essential in lymphoproliferation suppression and in the presentation of defective antigens (Rodrigues Júnior et al., 1992). TGF-β developed by adherent antigen-presenting cells from infected hamsters were involved in immunosuppression because when the *Leishmania* antigen-induced lymphoproliferative response was interrupted, a high level of TGF-ß was recorded in the cell culture supernatant (Rodrigues et al., 1998).

Further, *L. donovani* infection in hamster causes impairment in parasite antigen proliferation due to the inability of APCs to induce a specific T cell response, downregulation of protein kinase C and production of TGF-β leading to lymphocytes apoptosis (Rodrigues Júnior et al., 1992; Mookerjee et al., 2003; Banerjee et al., 2011). TGF-β secreted by macrophages is upregulated in infected hamsters. Neutralizing TGF-β with antibody reduced the lymphocyte apoptosis. The apoptosis of lymphocytes by TGF-β is mediated by the upregulation of tyrosine phosphatase activity (Banerjee et al., 2011). A protein LJM19 was identified that protected the hamsters from severe outcomes. The hamsters immunized with LJM19 displayed a low parasite burden, increased iNOS production, and a high IFN-γ/TGF-β ratio even after 5 months post-infection. Also, delayed-type hypersensitivity (DTH) associated with IFN-γ over-expression was observed in hamsters after 48 hours of biting of an uninfected sand fly. Activation of IFN-γ response at the site of a bite leads to protection in the viscera of immunized hamsters by direct killing of parasites or by priming the anti-parasite immune response (Gomes et al., 2008).

The hamsters infected *via* an intracardiac route with *L. donovani* amastigotes developed low parasite-specific antibodies while an increased serum immunoglobulin due to B cell polyclonal activation (Campos-Neto and Bunn-Moreno, 1982). The humoral response to VL in hamsters is mainly mediated by IgG. This was evident from a study that analyzed the quantity of IgG, IgA, and IgM antibodies in the hamster model of VL. The serum level of infected hamsters showed a six-fold increase in IgG2 as compared to uninfected hamsters. Similarly, the levels of IgG1 and IgA increased two to three-fold while the levels of IgG3 and IgM were comparatively the same in infected and uninfected hamsters (Melby et al., 2001). IgG deposit was observed in lung capillary walls and increased until day 30 post-infection and later declined. The parasite burden and anti-parasite antibody titer increased in the liver and spleen of infected hamsters (Mathias et al., 2001). This study suggested that antibodies are associated with increased lesions and disease severity in infected hamsters instead of imparting a protective immune response (Mathias et al., 2001). Although the hamster model shows significant similarity with human VL pathology, less focus has been given due to the unavailability of key reagents like antibodies and cytokines.

### Cytokine Response in Canine

In murine models, experimental data for VL in bulk is developed, but very little work has been reported on leishmaniasis using the dog as an animal model. Compared to mice and hamsters, the mechanism of the immune response to *Leishmania* infection in canines is not well documented yet due to the unavailability of standardized immunological reagents for the characterization of canine immunology (Carrillo and Moreno, 2009). However, the dog genome data bank has provided some information regarding DNA sequences of some cytokines (Loría-Cervera and Andrade-Narváez, 2014). Wild and domestic canines (dogs) are the primary reservoirs of the *L. infantum, which* causes zoonotic VL. In recent years, the dog model of VL is gaining importance for the investigation of immune response and identifying *Leishmania* antigens in canine visceral leishmaniasis (CVL) that are involved in protective immunity. Because of the few studies on CVL, interpreting the profile of cytokine expression in CVL is a tough challenge. Lack of appropriate clinical symptoms, secretion of a low amount of anti-leishmanial antibodies, less of a parasite burden, and good *in-vitro* lymphoproliferative response or positive DTH response to *Leishmania* skin antigens have been correlated with the defensive response in dogs (Barbiéri, 2006). Previously it has been reported that in CVL, the cellular immune response is linked with the overexpression of IL-2, IFN-γ, and TNF-α (Maia and Campino, 2012). Active disease, on the other hand, is characterized by a significant humoral response, specific parasite immunosuppression, and the occurrence of a full spectrum of clinical manifestations, whose severity in different tissues and organs is in line with the density of the parasite (Reis et al., 2006). Thus the pattern of cytokines observed in this condition is a mixed Th1/Th2 response (Santos-Gomes et al., 2002). The exposure of *L. infantum* infected macrophages to PBMCs isolated from dogs immunized with *L. infantum* antigens resulted in IFN-γ- and NO-mediated killing of parasites, thus inducing a protective immune response (Holzmuller et al., 2005). Further, the Th1-type cytokines, e.g., IL-2, IFN-γ, and TNF-α, induced NO production and diminished infectivity of *L. infantum* in immunized dogs (Panaro et al., 2001). However, the over-expression of IFN-γ mRNA in naturally infected dogs could be linked with humoral (IgG1) but not a cell-mediated immune response. On the other hand, infected dogs with substantially more serious symptoms, demonstrated over-expression of IL-4 (Quinnell et al., 2001).

Further, infected dogs also showed elevated levels of IL-10 and IFN-γ mRNA in the splenic macrophages, which manifest a balanced Th1 and Th2 cytokine production (Lage et al., 2007). Likewise, non-symptomatic naturally infected dogs also showed increased levels of IFN-γ, TNF-α, and IL-13 cytokines, which is an indication of mixed type Th1 and Th2 cytokines responses (Menezes-Souza et al., 2011). In *L. infantum* infected asymptomatic dogs, both Th1 and Th2 cytokines are secreted, but a predominant Th1 response aids protective immune response (Chamizo et al., 2005). Stimulation of PBMC with *Leishmania* antigen upregulated the production of IL-10, IL-6, IL-4, IL-8, IL-2, IFN-γ, and TNF-α. In healthy dogs, the expression of IL-4, IL-2, IFN-γ, and IL-10 was lower, while IL-6 was higher (Chamizo et al., 2005). IL-6 is suggested as a disease marker for canine VL (de Lima et al., 2007). Further, increased anti-leishmanial antibody titers (hypergammaglobulinaemia) in CVL are usually associated with high levels of IL-6 (de Lima et al., 2007). The antigen-stimulated PBMCs in asymptomatic dogs had higher IFN-γ and IL-4 levels than non-stimulated cells (Chamizo et al., 2005). Also, IL-12 in association with IL-15 aids the cellular immune response in CVL. In vitro stimulation of PBMCs isolated from VL infected canines with recombinant IL-12 and IL-15 upregulated T-bet expression and downregulated the expression of programmed cell death protein-1 (PD-1) in lymphocytes. These findings suggest a crucial role of IL-12 and IL-15 in canine VL (Costa and Gomes, 2020). In CVL, the mixed responses of Th1- and Th2-type cytokines have been described in peripheral blood mononuclear cells (PBMCs) of experimentally infected but asymptomatic dogs by the surge of IL-2, IFN-γ, and IL-10 mRNA levels. However, the overexpression of IFN-γ and IL-2 in asymptomatic dogs during *L. infantum* infection could not be linked to the overexpression of IL-10 (Chamizo et al., 2005).

### Cytokine Response in Non-human Primates

Non-human primates (NHPs) have similar anatomy, physiology, and close phylogenetic relationships with humans, so NHP could provide appropriate animal models for human VL. Several NHP models have been developed to study human VL. Various old world monkeys including sykes, baboons, as well as the Indian Langur monkey (*Presbytis entellus*) (Garg and Dube, 2006), and a few New World monkeys, including Aotus monkeys (Chapman et al., 1981; Chapman et al., 1983), squirrel monkeys (Chapman and Hanson, 1981), and marmosets (Marsden et al., 1981), are a well-established model for VL. Further, macaques are also reported to a good animal model for VL (Porrozzi et al., 2006). These animals developed a systemic disease and mimic the symptoms of human VL such as fever, diarrhea, frequent weight loss, anemia, hypergammaglobulinemia, and hepatosplenomegaly. Cytokine response in NHPs against the VL is not well documented. However, *L. major* infected Macaques developed Th1 mediated immunity by expressing IFN- γ, TNF-α, and IL-12 (Amaral et al., 2001; Gicheru et al., 2001; Freidag et al., 2003). In addition, an elevation in the number of splenic CD8^+^ T cells has also been demonstrated in macaque infected with *L. infantum* (André et al., 2020). During VL, IFN-γ plays an important role in parasite killing and elimination however, the importance of effector cytotoxic molecules not yet very clear. Interestingly, the cytotoxic molecule granulysin, which mimics the function of IFN-γ in the clearance of intracellular parasites, is reported to be absent in the mouse model (Dotiwala et al., 2016), but granulysin is reported to be well expressed in NHP models so this model could be a more accurate model of VL (Laforge et al., 2018).

### Cytokine Response in Human

In humans, *Leishmania* infection is typically subclinical and parasites can continue to survive through various escape mechanisms for the lifetime of the host (Dayakar et al., 2019). Various immune cells play a significant role in host defense during active VL for example, a drastic reduction in IL-8 secretion from neutrophils and reduced number of IFN-γ^+^ and IL-12^+^ eosinophils are observed in active VL patients; however, the number of IL-4**^+^** neutrophils and IL-10**^+^** eosinophils are reported to be augmented (Peruhype-Magalhães et al., 2005; Elshafie et al., 2011). Revival from VL is dependent entirely on T-cell immunity induction, particularly the Th1 response, which is primed by IL-12**^+^** DCs and macrophages (Nylén and Gautam, 2010). The importance of Th1 type cellular immune responses in protecting against *Leishmania* infection is well known in human leishmaniasis. In humans, there is strong evidence to suggest that IFN-γ is involved in regulating *Leishmania* infection. It has been reported that the *L. chagasi* infection is tackled by the elevated level of IFN-γ in *Leishmania* antigen-stimulated PBMCs from the blood of children from endemic regions, whereas children with the low level of IFN-γ susceptible to VL infection (Carvalho et al., 1992). These findings strongly suggest that the lack or lower production of IFN-γ is a marker of vulnerability to VL. It is quite possible that many mechanisms are likely to be involved in the inability to produce IFN-γ by lymphocytes during VL (Kima and Soong, 2013). The first possible mechanism is that the IFN-γ concentration may not be adequately wide or the period of the IFN-γ production may not be maintained sufficiently enough to reach other infected or distant bystander cells. Many *in vitro* studies at the single-cell level support this possibility (Vargas-Inchaustegui et al., 2008; Vargas-Inchaustegui et al., 2009; XIN et al., 2011; Macedo et al., 2012). According to the second possible mechanism, in bystander cells, IFN-γ receptor expression may not be high enough to react to IFN-γ. There have been records of impaired expression of the IFN-γ receptor in infected cells. (Ji et al., 2003). Another Th1 cytokine IL-8 is mainly associated with neutrophil infiltration in VL patients. The increased serum level of IL-8 in VL patients suggests the involvement of IL-8 in disease progression (Barral-Netto et al., 1991). In PBMC samples from pediatric VL, the frequency of IL-9**^+^**CD4**^+^**T cells was higher during infection, which gradually decreased upon the treatment, thereby implying the role of these cells in VL pathogenesis (Moravej et al., 2020).

It is well documented that during active human VL the production of IL-4 and IL-10 is elevated and production of IL-2 and IFN-γ is reduced (Murphy et al., 2001; Buxbaum and Scott, 2005; Thomas and Buxbaum, 2008; Bhowmick et al., 2009; Castellano et al., 2009). The lymphocytes of active VL patients are reported to express more IL-4 mRNA (Karp et al., 1993; Carvalho et al., 1994), and, similarly, the serum of VL patients has elevated levels of IL-4 (Zwingenberger et al., 1990). So far, there is no information that IL-4 is engaged in downregulating the Th1 type immune response in human leishmaniasis. For example, the *in vitro* addition of monoclonal antibody (mAb) to IL-4 did not resume the proliferative response of lymphocytes or the development of IFN-γ in *L. chagasi* stimulated PBMCs from VL patients (Carvalho et al., 1994). In subjects cured of leishmaniasis, IL-4 also did not inhibit lymphocyte proliferative response or IFN-γ production. VL-infected patients also showed an increased level of IL-10 in the bone marrow, lymph nodes, and PBMCs supernatant stimulated with *L. chagasi* (Ghalib et al., 1993; Karp et al., 1993; Carvalho et al., 1994). It has been documented that in VL patients, IL-10 neutralization stimulates the clearing of parasites (Gautam et al., 2011). In VL, PBMCs are also unable to produce IL-12 (Ghalib et al., 1995), and the development of IFN-γ and lymphocyte proliferative response restoration can be achieved by the addition of IL-12 (Ghalib et al., 1995; Bacellar et al., 1996). Another cytokine, IL-15, can induce Th1/Th2 proliferation. In *L. infantum* infected patients, the blood level of IL-15 was higher in infected individuals as compared to cured ones. Also, the secretion of IL-15 in response to *Leishmania* antigen was higher in PBMCs isolated from infected individuals as compared to uninfected and healed. Furthermore, IL-15 significantly increased IL-12 and reduced IL-4 secretion upon *in vitro* stimulation *Leishmania* antigen (Milano et al., 2002). Altogether these findings suggest the protective role of IL-15 in human VL.

The demonstration that IL-10 evades the effect of IL-12 in inducing IFN-γ production in *L. chagasi* stimulated PBMC from VL patients indicates that IL-10 is the main cytokine that leads *Leishmania* infection to visceral disease (Bacellar et al., 1996). IL-10 blocks the activation of Th1 cells by downregulating the production of IFN-γ and IL-12, thereby displaying a cytotoxic response. Furthermore, as IL-10 also prevents activation of macrophage, it reduces the *Leishmania* killing property of these cells (Bacellar et al., 2000). In humans, IL-10 and TGF-β have been shown to suppress healing responses and avoid host-parasite clearance (Buxbaum and Scott, 2005; Thomas and Buxbaum, 2008; Castellano et al., 2009). Patients suffering from VL have been reported to have increased IL-10 production (Bhowmick et al., 2009) along with increased IL-6 (Ramos et al., 2016). A decrease in the level of IL-10 in the treated patients indicates that IL-10 is a susceptibility factor for VL (Murphy et al., 2001; Saha et al., 2007). A complication of VL is another disease called post-kala-azar dermal leishmaniasis (PKDL). It is characterized by increased T-cell response resulting in the upregulation of both Th1 and Th2 cytokines after stimulation of PBMCs with parasite antigen. The plasma level of IL-10 is upregulated in PKDL patients, and the plasma and skin levels of IL-10 can be correlated with disease progression and severity (Katara et al., 2012; Zijlstra, 2016).

Furthermore, there is an important association between circulating antigen-specific TGF-β levels and parasite burden in VL patients, indicating its involvement in parasite proliferation and progression of disease in humans (Bhattacharya et al., 2016). The latest finding on human splenic aspirates indicates that blockade of IFN-γ and TNF-α elevates the production of IL-4, which is not responsible for replication of parasites, and IL-10 production. The biological role of IL-4 in the target organ of human VL remains an outstanding issue (Singh and Sundar, 2018). Moreover, another cytokine IL-6 is also linked with the severity and death during human VL, which is due to the inhibition of TNF-α in the early stage of infection and consequently by inhibiting the Th1 responses (Costa et al., 2013; Dos Santos et al., 2016). Further, during active VL, an increased level of IL-27 was also reported in human plasma and as compared to post-treatment tests, splenic mRNA levels of IL-27 and IL-21 were elevated in pre-treated biopsies (Ansari et al., 2011). IL-27 secretion enhances the response of Th1 but also weakens IL-17 development, which reduces the recruitment of neutrophils to target organs (Quirino et al., 2016). Furthermore, the reduced level of IL-1 has also been found to be associated with *L. donovani* infection of the human circulatory monocyte population (Reiner, 1987; Reiner et al., 1990). Likewise, during acute VL, human PBMCs failed to develop IL-1 in response to the stimulation of the *Leishmania* antigen *in vitro*. Although, IL-1 and TNF-α levels are typically recovered after anti-leishmanial therapy, which corresponds with clinical cure (Ho et al., 1992). Further, IL-3 also protects the host from VL. Together with macrophage colony-stimulating factor (M-CSF), granulocyte-macrophage colony-stimulating factor (GMCSF), and IFN-γ, IL-3 demonstrates the enhancing effect of oxidative burst stimulation and TNF-α secretion on human macrophages to prevent replication and development of the *Leishmania* (Elliott et al., 1989).

## Cytokine Production and T Cell Polarization in VL

The cellular immune response bridges the gap between innate and adaptive immune responses. The different T cell subsets play an important role in cytokine secretion during leishmaniasis. In VL, the CD4**^+^** T cells secrete pro-inflammatory cytokines such as TNF-α and IL-12, providing immunity against the parasite (Jawed et al., 2018); however, this is not observed in all conditions. In the hamster model of VL during the active phase of the disease, the splenic CD4**^+^** T cells displayed mixed expression of Th1 and Th2 cytokines (Medina-Colorado et al., 2017). Also, the splenic CD4^+^ T cells had upregulation of the PD-1 receptor. Blocking of PD-1 decreased arginase-1 production, decreasing the parasite burden in the spleen (Medina-Colorado et al., 2017). In another murine model of VL, the infection-induced IFN-γ secreting CD4**^+^** T cells are related to damage to bone marrow function (Pinto et al., 2017). Altogether these findings imply that the presence of a large population of CD4**^+^** T cells is not enough for a protective immune response against VL. The polarization of CD4**^+^** T cells for inducing an inflammatory response is crucial for anti-parasitic functions (Jawed et al., 2019). CD8**^+^** T cells are a different subset of T cells involved in protective VL immune response. This immune response is mainly mediated by IFN-γ, granzyme, and perforin secretion. The vaccine-induced CD8**^+^** T cells decrease organ parasite burden in a CXCL-10-dependent manner (Majumder et al., 2012). The recovered VL patients have upregulated CD8**^+^** T cell levels that aid granzyme-B-mediated parasite resistance (Kaushal et al., 2014). In human VL, there is an event of CD8**^+^** T cell exhaustion that decreases the anti-parasite activity mediated by CD8**^+^** T cells, thereby facilitating the parasite persistence and disease progression (Gautam et al., 2013).

The Th17 subsets of T cells also aid a protective immune response against VL. This protection is mainly mediated by the secretion of IL-17 and neutrophil infiltration (Gonçalves-de-Albuquerque et al., 2017). IL-1β, IL-6, and IL-23 primarily regulate the Th17 differentiation. However, the role of IL-17 in VL is contradictory. It aids the anti-parasitic activity by neutrophil infiltration. The VL patients with increased serum IL-17 levels displayed reduced parasite burden and rapid recovery (Tiwananthagorn et al., 2012). Also, DC stimulated with parasite antigen and peptidoglycan induces IL-17 secretion and aids protection from parasite infection (Jawed et al., 2016). On the contrary, IL-17 was also reported to increase susceptibility to *L. donovani via* downregulation of IFN-γ secretion and neutrophil migration (Terrazas et al., 2016). Thus, the detrimental effects of *Leishmania* infection are not solely contributed by IL-17, but other cytokines regulation of pathophysiology can reverse the effect (Jawed et al., 2019). Treg cells are extensively explored in experimental and human VL and are associated with increased VL susceptibility due to upregulated TGF-β and IL-10 levels (Rai et al., 2012). Treg cells induce IL-10 expression that, in turn, affects the IL-10 producing CD4**^+^** T cells, leading to further immune suppression in the host (Leveque et al., 2009). The interconversion between Treg and Th17 cells is IL-2 dependent. IL-2 aids increased Th17 cells and reduced Treg cells (Karmakar et al., 2012). Besides, another study reported that CD4**^+^** Foxp3**^+^** Treg cells increase the susceptibility to *Leishmania* infection (Tiwananthagorn et al., 2012).

## Discussion

Currently, the development of prophylactic and therapeutic measures has proven a challenging task due to the complex nature of the immune response. Effective parasite clearance can only be achieved with strong coordination of the innate and adaptive immune system. Cytokines are the major players that connect the bridge between cell mediate and humoral response needed for any successful therapy. Rodent models are being considered as primary testing models for screening of drug or vaccine candidates although they have certain disadvantages due to eliciting different immune responses compare to human beings against VL. Dogs and non-human primates are considered for secondary testing models, and their immune response closely resemble that of humans. It is important to understand how the host can respond to a particular infection. Immune response mediates by different cytokines may vary in different hosts which also help to establish the concept of the dichotomy between resistance and susceptibility. In the case of VL, the debate between resistance and susceptibility is still unsolved. Here, we tried to summarize cytokines response in different organisms of VL infection (**Table 1**). In the mouse model of VL, we looked up mainly the three most studied mice strain such as BALB/c, C57BL/6, and SV/129. VL in mice is chronic but not fatal, even the most susceptible mouse strain- BALB/c is also able to control the infection. The immune response in murine VL is organ-specific with the spleen being the initial site of infection. The parasites later migrate to the liver where, after 4 weeks, an effective Th1 immune response is triggered, resulting in parasite clearance. It has recently been reported that T cell cytokine response plays a crucial role to justify resistant vs. susceptible phenotypes in mice strains. IL-10, a Th2 cytokine promotes the VL infection in BALB/c at 8 weeks post-infection (Nylén and Gautam, 2010) while C57BL/6 mice did not upregulate IL-10 level. In contrast, the resistant strain SV/129 mice upregulate IL-10 expression after infection in CD4**^+^** T cells only 8 weeks post-infection. The role of the Thl/Th2 paradigm that is closely associated with resistance vs. susceptibility in VL infection is not completely understood (Wilson et al., 2005; Tripathi et al., 2007). BALB/c mice are prevalent in the Th2-like response while it is not in the case of C57BL/6 (Watanabe et al., 2004) or in SV/129 mice. The Syrian golden hamster (*Mesocricetus auratus*) is susceptible to VL infection and it was used to understand the mechanisms of immunosuppression. Elevated mRNA levels of Th1 cytokines were observed after the 1st week post-infection and, later on, as was a low level of IL-12 and IFN-γ transcript, while the Th2 cytokine IL-4 did not elevate in *L. donovani*-infected hamsters. However, IL-10 expression was increased during infection supporting disease progression. Remarkably, among all rodent models of VL infection, the Syrian hamster (*M. auratus*) closely resembles the clinicopathological features of human VL and unbiased immune response makes it the best rodent model. In contrast to the mouse model, in a hamster model of VL, the parasite replication is uncontrolled leading to death. The progression of disease occurs even after the activated Th1 response. The failure of APCs to trigger the antigen-specific T cells and apoptosis of T cells by TGF-β induction are major events in the failure of the protective immune response. Adding to this, the effector NO production of macrophages is also impaired in infected hamsters. Altogether, even after a prominent Th1 response, the disease becomes fatal due to the failure of key anti-parasitic elements.

Furthermore, due to limited research, understanding the cytokine expression profile in CVL is a difficult task. Protection is manifested through macrophage activation by IFN-γ and TNF-α to clear intracellular amastigotes through the L-arginine nitric oxide pathway. While the detectable level of IL-4 mRNA in infected dogs indicates disease progression (Quinnell et al., 2001). Also, a mixed cytokine profile of Th1 and Th2 was shown (Chamizo et al., 2005). Similarly, the immune response to the infection of VL in non-human primates is little understood, but they are often used as models of VL. IFN-γ, a key cytokine inhibiting the development of intracellular amastigotes in VL but the exact mechanism is still unclear.

The importance of protective Th1 immune response in experimental and human leishmaniasis is well established. In humans, the development of IFN-γ is correlated with infection control in *L. chagasi*-infected children. Further, lack of IFN-γ and increased level of IL-4 and IL-10 (Th2 cytokines) in antigen-stimulated PBMCs was also reported during VL. In addition, IL-12 restores the production of IFN-γ and improves the cytotoxic response. IL-10 suppresses IFN-γ secretion and anti-IL-10 mAb recovers the production of IFN-γ and lymphoproliferative response during *in vitro* condition. (Panaro et al., 2001; Gautam et al., 2011). Further other cytokines also play a promising role in VL e.g. IL-1, IL-3, IL-5, IL-6, IL-8, IL-9, IL-13, IL-15, IL-18, IL-23, and IL-27.

As discussed above, the Th1 cytokines especially IFN-γ are crucial for the protective immune response against human VL. Although experimental murine models of VL do not allow exact extrapolations with subclinical infection in humans they have been useful to identify genes and predict their functional roles in the protective immune response. Genetically resistant mice have the functional Slc11a1 gene, which is involved in macrophage activation (Blackwell et al., 2009). The Slc11a1 gene encodes a protein expressed on the membrane of infected macrophages and exerts an enhanced effect on iNOS expression and generation of NO, restricting intracellular *Leishmania* multiplication (Blackwell et al., 2001). In this context, visceral infection in BALB/c mice provides a good model for the evaluation of immunotherapy and vaccine candidates.

Recently, a newly emerging branch based on immunotherapy has been shown promising results to control various ailments including VL. For example, IL-15 could be a potential therapeutic agent in acute VL since it upregulates IL-12 and in combination with IFN-γ may increase the efficacy of conventional antimonial therapy for VL (reviewed in Dayakar et al., 2019). Similarly, targeting the IL-10 and other immunosuppressive factors by neutralizing antibodies could also demonstrate therapeutic benefits (reviewed in Singh and Sundar, 2014). Cytokines are important for immunotherapy against experimental and human VL so it is quite essential to explore their role in detail. The expression profile of various cytokines during disease progression as well as in host protection could give a clue for the development of new diagnostic tools and therapeutic measures against VL.

## Author Contributions

MS and PK collected the information and wrote the manuscript. US and SP helped to prepare the manuscript and in the modification of the text. MS and PK made the final draft of the manuscript. All authors contributed to the article and approved the submitted version.

## Funding

This work is supported by DBT–Ramalingaswami Re-entry grant BT/RLF/Re-entry/57/2017 to PK and DST-FIST grant SR/FST/LS- I/2018/131to Department of Zoology.

## Conflict of Interest

The authors declare that the research was conducted in the absence of any commercial or financial relationships that could be construed as a potential conflict of interest.
